# The prognostic value of TP53 mutations in oesophageal adenocarcinoma: a systematic review and meta-analysis

**DOI:** 10.1136/gutjnl-2015-310888

**Published:** 2016-01-05

**Authors:** Oliver M Fisher, Sarah J Lord, Dan Falkenback, Nicholas J Clemons, Guy D Eslick, Reginald V Lord

**Affiliations:** 1 Gastroesophageal Cancer Program, St Vincent's Centre for Applied Medical Research University of New South Wales, Sydney, New South Wales, Australia; 2 NHMRC Clinical Trials Centre University of Sydney, Sydney, New South Wales, Australia; 3 Department of Epidemiology and Medical Statistics, School of Medicine, University of Notre Dame, Sydney, New South Wales, Australia; 4 Department of Surgery, Lund University Hospital (Skåne University Hospital) and Lund University, Lund, Sweden; 5 Cancer Biology and Surgical Oncology Research Laboratory, Peter MacCallum Cancer Centre, Melbourne, Victoria, Australia; 6 Sir Peter MacCallum Department of Oncology, University of Melbourne, Melbourne, Victoria, Australia; 7 The Whiteley-Martin Research Centre, Discipline of Surgery, The University of Sydney, Sydney, New South Wales, Australia; 8 Department of Surgery, School of Medicine, University of Notre Dame, Sydney, New South Wales, Australia

**Keywords:** OESOPHAGEAL CANCER, META-ANALYSIS

## Abstract

**Objective:**

To clarify the prognostic role of tumour protein 53 (TP53) mutations in patients with oesophageal adenocarcinoma (OAC) as there is a need for biomarkers that assist in guiding management for patients with OAC.

**Design:**

A systematic review was conducted using MEDLINE, Embase, PubMed and Current Contents Connect to identify studies published between January 1990 and February 2015 of oesophageal cancer populations (with OAC diagnoses >50% of cases) that measured tumoural TP53 status and reported hazard ratios (HR), or adequate data for estimation of HR for survival for TP53-defined subgroups. Risk of bias for HR estimates was assessed using prespecified criteria for the appraisal of relevant domains as defined by the Cochrane Prognosis Methods Group including adherence to Grading of Recommendations, Assessment, Development and Evaluation and REporting recommendations for tumor MARKer prognostic studies guidelines, as well as assay method used (direct TP53 mutation assessment vs immunohistochemistry) and adjustment for standard prognostic factors. A pooled HR and 95% CI were calculated using a random-effects model.

**Results:**

Sixteen eligible studies (11 with OAC only and 5 mixed histology cohorts) including 888 patients were identified. TP53 mutations were associated with reduced survival (HR 1.48, 95% CI 1.16 to 1.90, I^2^=33%). A greater prognostic effect was observed in a sensitivity analysis of those studies that reported survival for OAC-only cohorts and were assessed at low risk of bias (HR 2.11, 95% CI 1.35 to 3.31, I^2^=0%).

**Conclusions:**

Patients with OAC and TP53 gene mutations have reduced overall survival compared with patients without these mutations, and this effect is independent of tumour stage.

**Significance of this study**

**What is already known on this subject?**

- Oesophageal adenocarcinoma (OAC) remains one of the few GI malignancies where molecular information is not taken into account to guide patient management.
- Current clinicopathological staging fails to accurately identify patients with OAC with good or poor prognosis.
- Recent genomic findings have identified a high tumour protein 53 (TP53) mutation rate in OAC, but the prognostic impact of this remains unclear due to conflicting reports in the literature.

**What are the new findings?**

- This is the first dedicated systematic review and meta-analysis regarding the prognostic impact of TP53 mutations in patients with OAC including 16 studies with >850 patients.
- We identify a significant negative prognostic impact of TP53 mutations on overall survival of patients with OAC.
- A greater prognostic effect can be corroborated in studies that report tumour-stage adjusted survival for OAC-only cohorts and are assessed at being at low risk of bias.

**How might it impact on clinical practice in the foreseeable future?**

- This study suggests that TP53 gene mutations have a clinically important negative prognostic impact on patients with OAC.
- In light of recent genomic findings highlighting a central role of this gene in OAC pathogenesis and drugs currently in development and testing that directly target this gene, a role for identifying TP53 mutations in OAC can be suggested.

## Introduction

The incidence of oesophageal adenocarcinoma (OAC) has increased faster than any other cancer since the 1970s in many Western countries with highest incidence rates found in Northern and Western Europe, Northern America and Oceania,^1–4^ with a greater than sixfold increase during the past three decades.^1^ ^5^ Population-based studies have observed consistent changes across different age and tumour stage groups, indicating that the rise of OAC incidence is a true increase and not an artefact of enhanced surveillance programmes.^1^ ^6^ Possible explanations for this increase include the increasing prevalence of obesity and Barrett's oesophagus (BO),^7^ well-recognised risk factors for OAC.

Fewer than half of the patients with a new diagnosis of OAC are eligible for curative treatment, and OAC continues to have one of the highest cancer case-fatality rates with population-based 5-year survival rates typically around 15%.^8^ ^9^ For those patients referred to curative treatment, usually by neoadjuvant chemotherapy or chemoradiotherapy (CRTx) followed by oesophagectomy, 5-year survival rates are still generally <45%.^10^ In contrast to other malignancies, such as breast and colon cancer, where the incorporation of molecular information has become part of routine practice for therapeutic stratification,^11^ current treatment algorithms for OAC still depend on only imaging and histological assessments to determine disease stage and grade to guide treatment and help classify prognosis.

The tumour-suppressor gene tumour protein 53 (TP53) (National Centre for Biotechnology Information gene ID: 7157), which encodes the p53 protein and is sometimes called ‘the guardian of the genome’,^12^ is one of the most frequently mutated and studied genes in human cancers.^13^ The p53 protein plays multiple functions in regulating cell cycle progression and apoptosis, autophagy, differentiation and senescence, as well as DNA repair functions, and also exerts effects on cell metabolic pathways and cytokines.^14^ Substantial efforts have been made to study the effect of TP53 mutations on prognosis for patients with cancer. Furthermore, as most chemotherapeutic agents act by inducing DNA damage,^15–17^ the predictive effect of TP53 gene mutations on therapeutic response has also been explored.^18^

Recent large-scale whole-genome and whole-exome sequencing studies have shown that both OAC and dysplastic BO harbour a very high TP53 mutation rate of up to 70%,^19–21^ indicating a central role for this gene in OAC pathogenesis. This finding raises the question of whether the 30% of patients harbouring wild-type TP53 may thus have a different underlying tumour biology that may impact patient outcome.

We aimed to resolve the existing uncertainty regarding the prognostic value of TP53 for staging OAC by conducting a systematic review and meta-analysis of all published data with subgroup analysis of studies assessed as low risk of bias, and studies using direct TP53 gene mutation analysis techniques to determine TP53 mutation status, since these are the most accurate methods for determining tumoural TP53 mutations.

## Materials and methods

### Search strategy and study eligibility criteria

The electronic bibliographic databases MEDLINE, Embase, PubMed and Current Contents Connect were searched to identify eligible studies published between January 1990 and 8 February 2015 using MeSH terms and text words for adenocarcinoma*, o/esophagus* or o/esophageal*, TP53* or p53* or 17p13* or 17p*. In an attempt to minimize the risk of publication bias, conference abstracts and proceedings were searched through Web of Science, Embase and Scopus using the terms o/esophagus* and p53*. Further, the following major GI and oncological conferences were searched for relevant reports: Digestive Disease Week (DDW; by screening the DDW website and supplementary material of the journal *Gastroenterology*), American Society for Clinical Oncology (ASCO; by searching the ASCO library and *Journal of Clinical Oncology* supplementary material) and American Association for Cancer Research (AACR; searched through their webportal of all AACR conference proceedings) from 1990 to 2015.

Two reviewers (OMF and DF) scanned the search results (title and abstract) and retrieved full text publications using the criteria outlined below to identify eligible studies. Reference lists of relevant studies identified from the search including reviews were further screened to identify studies that may not have been identified by the strategy outlined above.

Study inclusion criteria were prospective or retrospective clinical studies of OAC populations that assessed TP53 mutation status and/or p53 expression in primary tumours, and compared overall survival for TP53 mutation versus TP53 non-mutation subgroups with calculation of HR and 95% CIs, or reported adequate data for their estimation. To include all available data, we also included studies of oesophageal cancer cohorts that included patients with squamous cell carcinoma if at least ≥50% of the patient cohort had a diagnosis of OAC.

Study exclusion criteria were studies of TP53 DNA germline mutations or autoantibody detection in blood; and reports available in abstract form only that did not report adequate information to determine study eligibility or to assess study methods for risk of bias.

If studies did not report sufficient data to calculate HRs or in case of missing/unclear data, the corresponding author was contacted by email to request this information. If the same research unit (identified from author names and institution) published multiple reports with overlapping patient recruitment time periods, HR estimates were extracted from the most recent publication with the largest patient numbers to avoid duplication of data.

### Data extraction

Three investigators (OMF, DF and NJC) reviewed eligible studies and extracted the following variables into a standardised data extraction form: author's name; publication year; country where study was conducted; number of patients included and general patient demographics; tumour histology (number and proportion of OAC tumours included); treatment modality (surgery alone, neoadjuvant or adjuvant CRTx followed by surgery); tissue specimen type (surgical specimen vs endoscopic biopsy); TP53 assay methods (TP53 gene sequencing, single-strand confirmation polymorphism (SSCP), immunohistochemistry (IHC), and type of antibody, dilution for IHC); criteria or cut-point used to define TP53 mutation status for the survival analysis; study prevalence of TP53 ‘mutation’ and ‘non-mutation’ subgroups; median survival of all patients and by TP53 mutation status; unadjusted and adjusted HR with 95% CI and corresponding p values where available. For consistency and to facilitate further quantitative analyses, the authors’ definitions for TP53 ‘mutations’ were used for studies performing only IHC as the respective studies did not use uniform staining classification criteria. As such, nuclear p53 protein overexpression was interpreted to represent TP53 mutations by all authors of the included studies, although loss of p53 protein expression has also been associated with tumoural TP53 gene mutations.^22^ ^23^ This staining pattern was not reported and/or interpreted in such a manner in any of the included studies.

### Risk of bias assessment, subgroup and sensitivity analyses

All studies were assessed for risk of bias for the study estimate of the impact of TP53 on survival by appraising six domains (study participation, biomarker measurement, outcome measurement, confounding measurement and account, participant attrition, analysis method) using prespecified criteria adapted from the Grading of Recommendations, Assessment, Development and Evaluation (http://www.gradeworkinggroup.org),^24^ REporting recommendations for tumor MARKer prognostic studies (REMARK)^25^ and from Hayden *et al*'s^26^ (Cochrane Prognosis Methods Group) guidelines for quality appraisal for prognostic studies. Risk of bias for each domain was graded as high, low or unclear based on assessment of each criterion. The overall risk of bias for the study was assessed as high if one or more of the domains was assessed as high risk of bias as recommended by the Cochrane Collaboration.^27^

Assessment of the risk of bias for different methods of assessing TP53 mutation status was informed by data regarding the analytical validity of different methods reported in the International Agency for Research on Cancer (IARC) TP53 mutation database (R17)^13^ (as summarised in online supplementary file 1). Studies performing TP53 gene sequencing or direct assessment of TP53 gene mutations were assessed as being at low risk of biomarker measurement bias compared with studies performing only IHC analysis, based on estimates from the IARC TP53 mutation database that approximately 27% of all TP53 mutations stain as false negatives in OAC using IHC (see online supplementary figure S2). Studies that did not adjust for tumour stage to assess the independent impact of TP53 mutation status on patient survival were classified as high risk of bias.

10.1136/gutjnl-2015-310888.supp1 Supplementary materials

### Statistical analysis

To estimate the effect of TP53 mutation status on OAC survival, we calculated a pooled HR and 95% CI using the generic inverse variance method. If the HR was not reported, it was estimated from the corresponding Kaplan–Meier curves using the Parmar method.^28^ ^29^ If the SE was not reported, it was estimated from the 95% CI.

Because different TP53 mutation analysis methods were used across studies, we expected heterogeneity in study estimates of the TP53 mutation effect on survival, and thus we applied a random-effects model to estimate the HR.^30^ Heterogeneity was tested using Cochran's Q statistic, with p<0.1 indicating heterogeneity. The degree of heterogeneity was quantified using the I^2^ statistic.^31^ Meta-regression was performed to inspect possible sources of interstudy heterogeneity.^32^ Study level factors that may modify the prognostic effect of TP53 were included as covariates if they were present in ≥10 of the included studies.^27^

Sensitivity analysis was performed to assess the impact of tumour histology and assay type on survival by repeating the pooled HR analysis in the following subgroups: (i) OAC-only versus OAC-mixed study populations; (ii) IHC versus direct TP53 gene mutation analyses; and (iii) studies assessed as having low versus high risk of bias. Differences between subgroups were assessed with a test for interaction.^33^ In order to estimate the prognostic value of TP53 independent of stage, a sensitivity analysis was also performed of studies reporting HR adjusted by stage. Publication bias was quantified using the Egger's regression model and visualised using funnel plot analyses.^34^

Descriptive statistics as well as quantitative analysis of the IARC TP53 mutation database to guide risk of bias assessment were performed using R statistical software^35^ and the *ggplot2* package.^36^ Meta-analyses of HR estimates were performed using Review Manager (RevMan) V.5.2 software (Copenhagen: The Nordic Cochrane Centre, The Cochrane Collaboration, 2012) with meta-regression performed using the packages *metafor* and *forestplot* for R.^37^ ^38^

## Results

### Study characteristics

**Figure 1:**
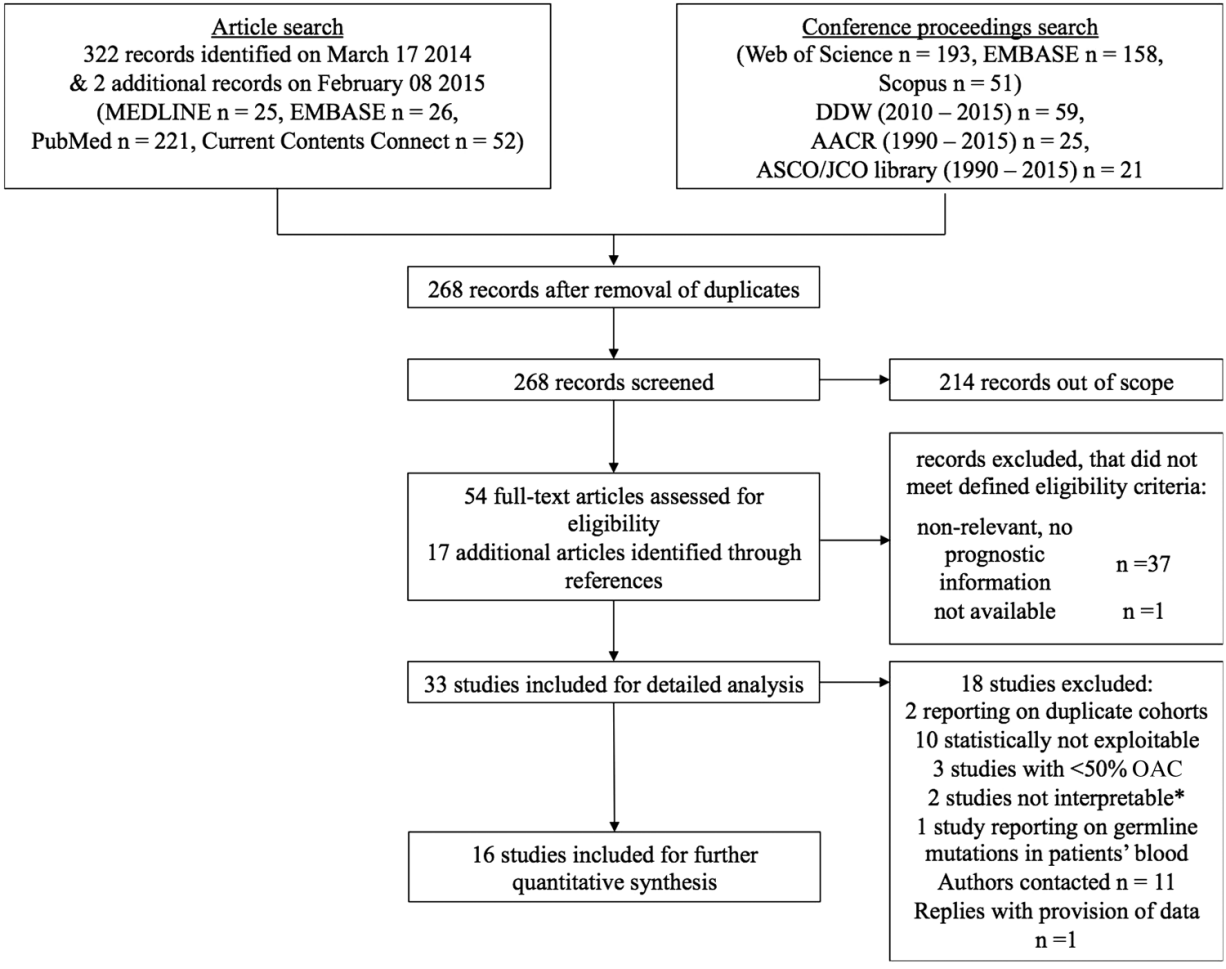
Preferred Reporting Items for Systematic Reviews and Meta-Analyses (PRISMA) flow chart of study identification process. *One study provided sufficient information for statistical extraction; however, interpretation of data was not possible due to inconsistent definition of protein 53 (p53) mutation status in survival analysis, in as much that p53 positive denoted a change from p53 positivity pre-radiochemotherapy to p53 negative post-chemoradiotherapy. One study only presented final survival data on loss of heterozygosity at chromosome 17q, which does not contain the tumour protein 53 (TP53) gene. AACR, American Association for Cancer Research; ASCO, American Society for Clinical Oncology; DDW, Digestive Disease Week; JCO, Journal of Clinical Oncology.

The search strategy yielded 323 studies, of which 16 met our eligibility criteria (figure 1). Study characteristics are summarised in table 1. Eleven studies included pure OAC cohorts^39–49^ and five studies included mixed histological cohorts,^50–54^ in which the percentage of OAC cases in the study data ranged from 56%^54^ to 81%.^53^ Overall, the 16 studies totalled 1211 patients including 986 OAC, with survival data reported for 888 patients. The number of patients with survival data in each study ranged from 16 to 142 (median 50) with a median number of OAC tumours of 53 (range 20–142) per study.

**Table 1:** Baseline characteristics of included studies

Author	Year	Country	N	N in survival analysis	Number of patients with OAC (% total)	Only curative surgery in survival analysis?	Specimen type	CRTx?	Neoadjuvant CRTx?	Analysis method	IHC antibody	Dilution	Percent ‘mutated’	HR estimation method	Multivariable analysis performed and reported in original paper?
Fléjou^39^	1993	France	62	62	62 (100)	Yes	Surgery	NR	NR	IHC	DO7 (Dako) and PAb1801	NR	66	Extrapolated	No
Duhaylongsod^40^	1995	USA	42	40	42 (100)	Yes	Surgery	Yes	Yes	IHC	PAb1801 (Oncogene Science)	NR	79	Extrapolated	No
Sauter^48^	1995	USA	24	16	24 (100)	Yes	Biopsies	Yes	Yes	IHC	PAb1801 (Oncogene Science)	1 μg/mL (?)	50	Extrapolated	No
Wu^41^	1998	USA	92	90	92 (100)	Yes	Surgery	Yes	Yes	LOH+IHC	DO7 (Dako)	100	57	Reported in text	Yes
Ribeiro^50^	1998	USA	42	35	31 (74)	Yes	Surgery	Yes	Yes	Sequencing	–	–	40	Reported in text, 95% CIs extrapolated	Yes
Soontrapornchai^49^	1999	Australia	135	51	135 (100)	No	Biopsies	Yes	Yes	SSCP	–	–	36	Reported in text	No
Schneider^43^	2000	Germany	59	49	59 (100)	Yes	Biopsies	Yes	No	Sequencing	–	–	44	Reported in text	Yes
Ireland^42^	2000	USA	37	22	37 (100)	Yes	N/A	No	No	Sequencing	–	–	49	Calculated from raw data	No
Aloia^51^	2001	USA	61	61	44 (72)	Yes	Surgery	No	No	IHC	#1801 (Biogenex)	200	67	Reported in text, 95% CIs extrapolated	Yes
Gibson^52^	2003	USA	54	46	41 (76)	Yes	Biopsies	Yes	Yes	Sequencing	–	–	63	Reported in text	Yes
Falkenback^44^	2008	Sweden	54	54	54 (100)	Yes	Surgery	No	No	IHC	DO7	300	60	Calculated from raw data	No
Madani^45^	2009	Canada	142	142	142 (100)	Yes	Surgery	No	No	Sequencing+IHC	DO7 (Dako)	50	47	Reported in text	Yes
Cavazzola^47^	2009	Brazil	46	38	46 (100)	Yes	Surgery	No	No	IHC	DO7 (PAb1801 Sigma)	100	52	Reported in text	Yes
Lehrbach^46^	2009	Brazil	75	75	75 (100)	Yes	Surgery	No	No	IHC	DO7 (Novocastra)	NR	60	Extrapolated	No
Fareed^53^	2010	UK	245	66	83 (81)	Yes	Surgery	Yes	Yes	IHC	DO7? (Vector Labs)	50	30	Extrapolated	No
Kandioler^54^	2014	Austria	36	36	20 (56)	No	Biopsies	Yes	Yes	Sequencing	–	–	50	Reported in text	Yes

CRTx, chemoradiotherapy; IHC, immunohistochemistry; LOH, loss of heterozygosity; NR, not reported; OAC, oesophageal adenocarcinoma; SSCP, single-strand confirmation polymorphism.

Half of the studies (n=8) assessed TP53 mutation status by IHC,^39^ ^40^ ^44^ ^46–48^ ^51^ ^53^ one study assessed TP53 mutations through 17p/17p.13 loss of heterozygosity (LOH),^41^ one through SSCP^49^ and the remaining six studies^42^ ^43^ ^45^ ^50^ ^52^ ^54^ performed TP53 gene sequencing to determine the presence of mutations. Using these methods, a median of 55% (range 33–79%) of all tumours and 57% of all OACs (range 33–79%) were classified as harbouring TP53 mutations.

The clinicopathological variables and survival times reported in the included studies are summarised in online supplementary table S1. Briefly, 10 studies provided information on pathological T-stage,^39^ ^42^ ^44–47^ ^50^ ^51^ ^53^ ^54^ 13 studies on pathological N-stage,^39^ ^40^ ^42^ ^44–51^ ^53^ ^54^ 8 studies on metastatic status^42^ ^44^ ^46^ ^47^ ^49^ ^50^ ^53^ ^54^ and 10 described the final tumour differentiation grade.^39^ ^42^ ^43^ ^45^ ^47–50^ ^52^ ^54^ Approximately half the studies reported an overall staging of patients according to Union for International Cancer Control (UICC) or American Joint Committee on Cancer (AJCC) criteria (n=9),^39^ ^41^ ^43–47^ ^50^ ^51^ and 5 studies described resection margin status.^43^ ^45^ ^47^ ^51^ ^54^ In the 14 studies^39–42^ ^45–51^ ^53^ ^54^ that reported survival time data for biomarker-defined patient subgroups, the median survival time for patients assessed as having mutated TP53 was 18.9 months (n=488) compared with 26.2 months for patients with non-mutated TP53 (n=423).

HRs were reported in nine studies and extrapolated from five studies. In addition, individual patient data were available for two studies to calculate tumour stage-adjusted HR and 95% CIs^42^ ^44^ (table 1). One of these studies^42^ included six subcardia cancers and had complete survival information on 22 patients, all with OAC cancers. One of the patients in this study was excluded from the survival analysis due to an early, postoperative mortality.^42^ The other study^44^ included survival data for all 60 patients with OAC.

Four studies^41–43^ ^54^ were assessed as being at a low risk of bias, and 12^39^ ^40^ ^44–53^ studies were assessed as being at high risk of bias (table 2). Funnel plot analyses did not reveal substantial publication bias (see online supplementary figure S3).

**Table 2:** Risk of bias assessment

Reference	Patient inclusion/exclusion criteria clearly defined	Patient treatment clearly characterised	Specimen characteristics	Adequate detection method of TP53 mutation	Study design	Study/statistical methods	Presentation (explanation of dropouts, number of events)	Reporting of basic demographic characteristics	Comparison of marker to standard prognostic variable	Univariate and/or time-to-event data presentation	Multivariable analysis adjusting for standard prognostic factors and adequate reporting hereof	Other potential sources of bias	Risk of bias
Fléjou *et al*^39^	Low	Low	Low	High	Low	Low	Low	Low	Low	Low	High	Low	High
Duhaylongsod *et al*^40^	Low	Low	Unclear	High	Low	High	Low	High	High	Low	High	Low	High
Sauter *et al*^48^	Low	Low	Unclear	High	Low	Unclear	Low	Unclear	Unclear	Low	High	High	High
Ribeiro *et al*^50^	Low	Low	Low	Low	Low	Low	Low	Low	Low	Low	High	Unclear	High
Soontrapornchai *et al*^49^	Low	Low	Low	Low	Unclear	Low	Low	Low	Low	Low	High	Low	High
Wu *et al*^41^	Low	Low	Low	Low	Low	Low	Low	Low	Low	Low	Low	Low	Low
Ireland *et al*^42^	Low	Low	Low	Low	Low	Low	Low	Low	Low	Low	Low	Low	Low
Schneider *et al*^43^	Low	Low	Low	Low	Low	Low	Low	Low	Low	Low	Low	Low	Low
Aloia *et al*^51^	Low	Low	Low	High	Low	Low	Low	Low	Low	Low	Unclear	Low	High
Gibson *et al*^52^	Low	Low	Low	Low	Low	Low	Low	Low	High	Low	High	High	High
Falkenback*et al*^44^	Low	Low	Low	High	Low	Low	Low	Low	Low	Low	Low	Low	High
Cavazzola *et al*^47^	Low	Low	Low	High	Low	Low	Low	Low	Low	Low	Low	Low	High
Lehrbach *et al*^46^	Low	Low	Low	High	Low	Low	Low	Low	Low	Low	High	Low	High
Madani *et al*^45^	Low	Low	Low	Low	Low	Low	Low	Low	Low	Low	High	Unclear	High
Fareed *et al*^53^	Low	Low	Unclear	High	Low	Low	Low	Low	Low	Low	High	Low	High
Kandioler *et al*^54^	Low	Low	Low	Low	Low	Low	Low	Low	Low	Low	Low	Low	Low

### Overall analyses

**Figure 2:**
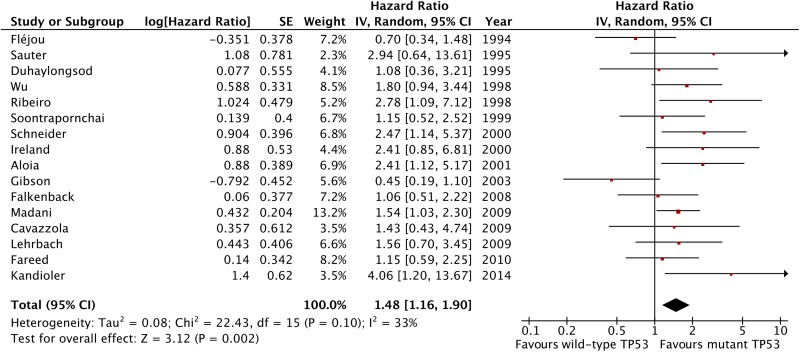
Forest plot of the effect of tumour protein 53 (TP53) mutation status on survival, all 16 included studies.

**Figure 3:**
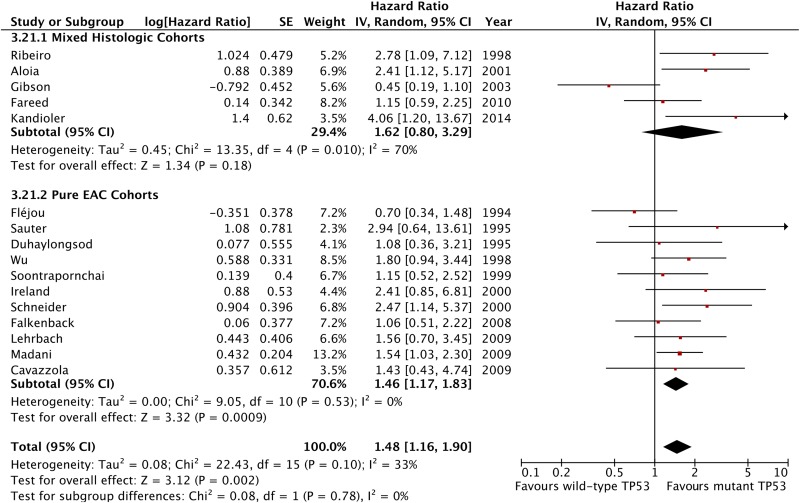
Forest plot of the effect of tumour protein 53 (TP53) on survival stratified by tumour histology included in studies.

The meta-analysis of data from all 16 included studies showed that TP53 mutation is associated with a statistically significant negative effect on patient overall survival with an HR 1.48 (95% CI 1.16 to 1.90, p=0.002, n=888 patients; figure 2) with low-moderate heterogeneity across studies that was not statistically significant (I^2^=33%, p heterogeneity=0.1). The analysis of studies including pure OAC patient cohorts showed similar results with low heterogeneity (HR 1.46, 95% CI 1.17 to 1.83, p=0.0009, n=11 studies and 644 patients, I^2^=0%, p for heterogeneity =0.53, p for interaction=0.78; figure 3).

### Subgroup and sensitivity analyses

**Figure 4:**
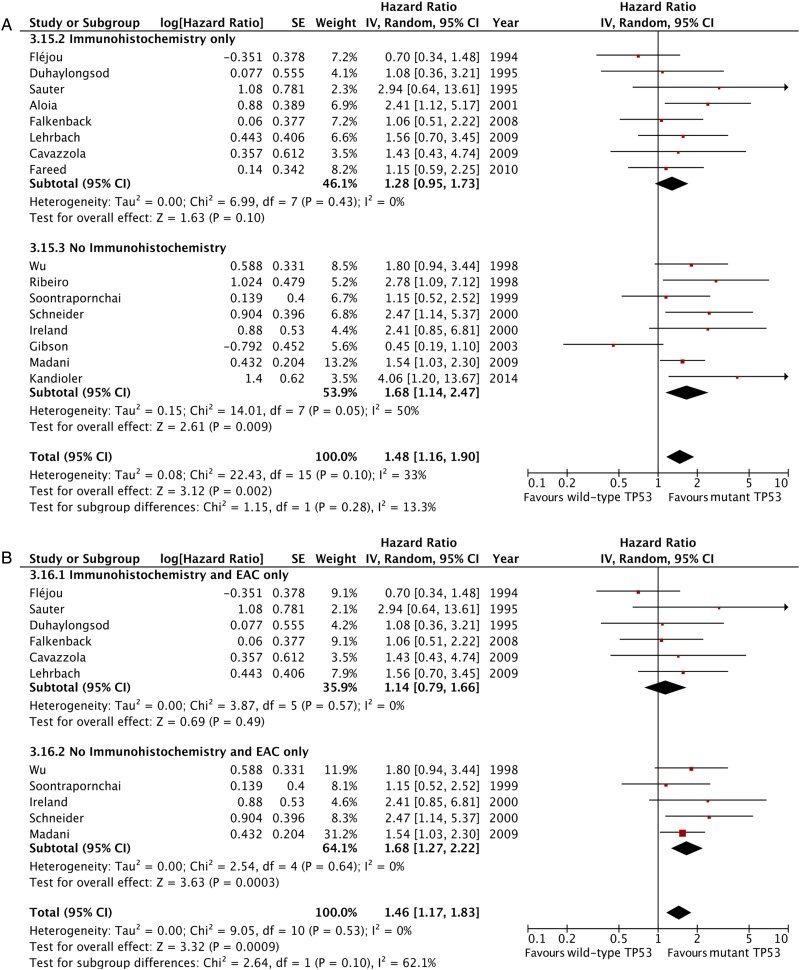
Forest plot of the effect of tumour protein 53 (TP53) on patient survival stratified by TP53 analysis methodology, including all studies (A) and only those studies with pure oesophageal adenocarcinoma cohorts (B).

The effect of TP53 mutation status on survival appeared to be smaller among studies performing IHC (pooled HR 1.28, 95% CI 0.95 to 1.73, p=0.10, 8 studies, 417 patients, I^2^=0%) compared with studies performing direct TP53 gene assessments (sequencing and SSCP) or LOH analyses (HR 1.68, 95% CI 1.14 to 2.47, p=0.009, 8 studies, 471 patients, I^2^=50%, figure 4A). However, this difference was not statistically significant (p for interaction=0.28). This finding was similar in studies including OAC only cohorts (figure 4B).

The effect of mutant TP53 on patient overall survival was larger in studies that had adjusted their analyses for tumour stage (HR 1.95, 95% CI 1.41 to 2.66, p≤0.0001, 7 studies, 430 patients, I^2^=0%; see online supplementary figure S4) compared with the estimates from studies that reported unadjusted risk estimates (HR 1.22, 95% CI 0.88 to 1.70, p=0.24, 9 studies, 458 patients, I^2^=38%). This difference was borderline statistically significant (p for interaction=0.05). A similar effect was seen in the subset of studies containing pure OAC cohorts (see online supplementary figure S4).

**Figure 5:**
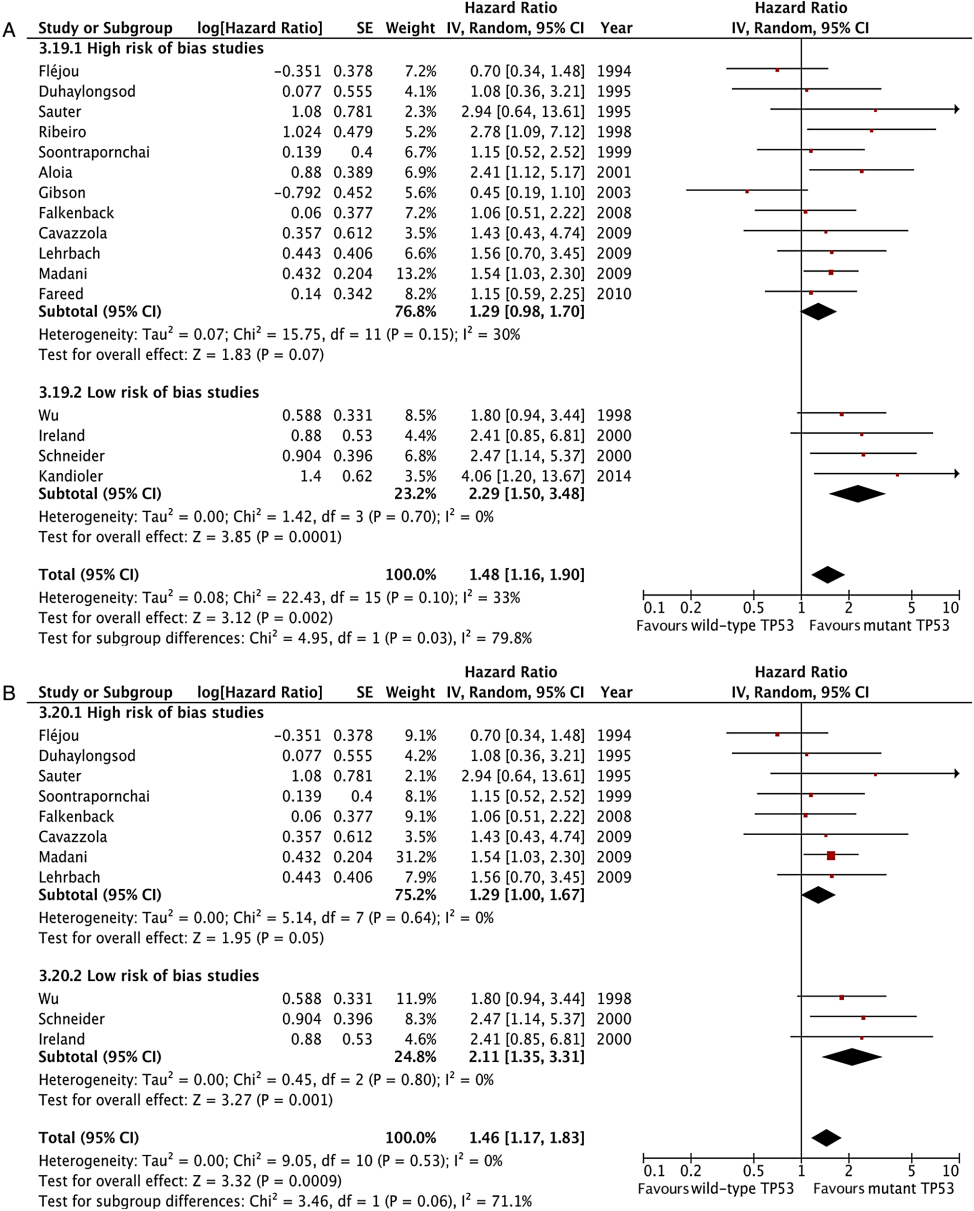
Forest plot of the effect of tumour protein 53 (TP53) on survival stratified by risk of bias assessment including, all studies (A) and only including studies with pure oesophageal adenocarcinoma cohorts (B).

The prognostic effect of TP53 mutations was also significantly larger in the subset of four studies assessed as low risk of bias (HR 2.29, 95% CI 1.50 to 3.48, p=0.0001, 197 patients, I^2^=0%; figure 5A), compared with those assessed as high risk of bias (HR 1.29, 95% CI 0.98 to 1.70, p=0.07, 691 patients, I^2^=30%, p for interaction=0.03). This effect size was similar in the three studies with low risk of bias that contained pure OAC cohorts (HR 2.11, 95% CI 1.35 to 3.31, p=0.001, n=161 patients, I^2^=0%; figure 5B).

### Exploratory subgroup and sensitivity analyses

Subgroup analysis of studies that determined TP53 status using gene sequencing also showed a negative prognostic impact of mutant TP53 on patient survival (HR 1.80, 95% CI 1.05 to 3.08, p=0.02, 6 studies, 330 patients, I^2^=62%; p for heterogeneity=0.02, see online supplementary figure S5A); however, significant interstudy heterogeneity was noted. Sensitivity analysis indicated that this was driven by the study by Gibson *et al*^52^—the only study with an HR <1. Removing this study provided an HR of 1.95 (95% CI 1.44 to 2.65, p <0.0001; 5 studies, 284 patients, I^2^=0%; see online supplementary figure S5B). Findings were consistent when the same subgroup analysis was performed for only those TP53 gene sequencing studies including pure OAC cohorts (HR 1.76, 95% CI 1.26 to 2.47; p=0.0009, 3 studies, 213 patients, I^2^=0%; see online supplementary figure S5C). A summary of the results of other exploratory subgroup analyses can be found in online supplementary table S2.

### Metaregression: potential sources of interstudy heterogeneity

Of the 14 inspected study covariates, only the adjustment of HR estimates for standard prognostic variables (change log HR 0.46, 95% CI 0.01 to 0.91; p=0.04) and the study appraised as being at low risk of bias (change log HR 0.58, 95% CI 0.06 to 1.10; p=0.03) were significant sources of heterogeneity (see online supplementary figure S6).

## Discussion

This study indicates that mutated TP53 negatively impacts overall survival in patients with OAC, independent of tumour stage. This effect is estimated as a relative increase in hazard of death of 48%, with up to a 211% increase when studies of mixed histology cohorts or high risk of bias are excluded. This corresponds to a reduced survival time of approximately 7 months based on median survival from the included studies. This effect size is similar to the difference in median survival of stage IIIA compared with stage IIIC OAC.^55^

To our knowledge, this is the first comprehensive systematic review and meta-analysis of the prognostic impact of TP53 mutations in patients with OAC that includes an assessment independent of tumour stage. Two previous systematic reviews and meta-analyses of various prognostic biomarkers in OAC have included an analysis of TP53 mutations.^56^ ^57^ Both studies reported similar significant negative effect estimates on patient survival with two^57^ and five^56^ primary studies in their respective analyses, but did not consider potential confounders such as tumour stage or TP53 mutation analysis methods. As such, with 16 studies and >800 patients, our study is the largest and most extensive analysis of the effect of TP53 mutations on OAC patient survival.

This study is timely because recent whole-genome sequencing studies have shown a high mutation rate of TP53 in OAC.^19^ ^20^ With whole-genome sequencing technologies still not used in clinical practice for personalised cancer treatment, targeted genomic approaches remain a valid area of investigation.^58^

One potential explanation for earlier conflicting results for the prognostic significance of TP53 status in OAC is the use of different and potentially less accurate assay methods for TP53 mutation detection. Our study identified considerable variability in IHC methods across the included studies, such as the use of different antibodies, antibody dilutions and variable scoring systems for immunopositivity. Further, none of the included studies used loss of p53 expression as a method for interpreting the presence of TP53 gene mutations.^22^ ^23^ These technical variations, combined with the knowledge that not all TP53 mutations lead to accumulation of mutant protein, and that wild-type p53 protein can be overexpressed, leading to either false negative or false positive staining results,^59^ may explain why IHC has been regarded as a less adequate analysis method in other cancers.^22^ In our subgroup analysis by assay type, the prognostic effect of TP53 appeared to be smaller in studies that used IHC versus other methods, particularly for mixed histology populations; however, a test for interaction was not statistically significant. We were also not able to demonstrate that the use of IHC contributed to interstudy heterogeneity in our meta-regression analysis. Nevertheless, with increased knowledge about TP53 mutation variants^60^ and more standardised and economical targeted gene sequencing technologies available,^61^ our results support the validity of using direct TP53 mutation status as a prognostic biomarker in OAC and raise the possibility that this provides improved prognostic classification than IHC consistent with findings in other cancers.^22^

Recent genomic studies have found that TP53 gene mutations remain the most common genetic alteration in both OAC^19^ ^20^ and its precursor lesion BO with dysplasia,^21^ with wild-type TP53 estimated to be present in only approximately 30% of OAC tumours. These findings suggest that the mutation frequency of TP53 may be underestimated in all studies included here, even those performing TP53 gene sequencing. Most studies only sequenced exons 5–8, whereas whole-genome sequencing (which includes the sequencing of intronic and intergenic regions) has been shown to be more accurate at detecting mutations, even within exonic regions.^22^ ^62^ Further research using current sequencing technologies will be needed to assess whether the overall prognostic impact of TP53 mutation status is larger or smaller than the present study estimate, and to determine whether the prognostic effect of TP53 mutations varies by type of gene mutation. While our subgroup analysis of only TP53 gene sequencing studies showed a pronounced effect of TP53 mutations on OAC patient survival, this pooled analysis displayed substantial heterogeneity. This was largely caused by one study^52^ that reported a non-significant survival advantage for TP53 mutations. Although this study included sequencing data from pretreatment biopsies of mainly patients with OAC, no information is provided on patients’ pathological AJCC/UICC tumour stage, which to date remains the most accurate predictor of patient prognosis.^55^ Study patients were enrolled from two experimental, prospective studies assessing the effect of two similar neoadjuvant CRTx regimens. Patients were only staged clinically and the HR estimation could not be adjusted for pathological tumour stage, because of which we assessed the estimate as being at high risk of bias. A sensitivity analysis performed to exclude this study suggests that gene sequencing-defined TP53 mutations are associated with an almost twofold mortality risk (HR 1.95, 95% CI 1.44 to 2.65).

The two main limitations of this study that may affect the validity of our findings are the quality of the primary studies and data limitations to explore potential confounders. First, more than half of the studies were assessed as high risk of bias based on criteria defined by Hayden *et al,*^63^ an aspect also identified as a significant contributor to interstudy heterogeneity. Equally, the lack of adjustment of HR estimates for standard prognostic criteria, as recommended in current biomarker reporting guidelines,^25^ was also a significant contributor to heterogeneity. These methodological flaws may lead to an underestimate of the actual effect size, as suggested by our subgroup analyses. Second, we were unable to conduct analyses to investigate the impact of potentially relevant factors (such as the inclusion of Siewert Type III or proximal gastric cancers or patient smoking status) because of the lack of data reported in the original publications. Other concerns may be how the inclusion of mixed patient populations, different staging systems and treatment regimens may impact the present findings. However, our meta-regression did not identify these factors as potential sources impacting overall effect estimates. Further, the key finding of TP53 negatively affecting patient prognosis persisted across all pooled and subgroup analyses, despite our comprehensive methodological approach, which accounted for sources of confounding, bias and interstudy heterogeneity. But most of the studies only included patients from surgical series, potentially limiting the generalisability of our findings. Despite almost 70% of the studies (n=11) having AJCC stage IV patients in their analysis, only nine of these studies also included such patients in their p53-stratified survival analysis. With the median percentage of stage IV patients in such studies being limited to 5.5%, the generalisability of our findings to patients with OAC with more advanced stages of disease that do not permit curative treatment is limited. Finally, despite conducting an extensive search strategy including searching conference abstracts and presenting a funnel plot that excludes major asymmetry, we cannot eliminate publication bias as a possible explanation of our results.

Further studies are warranted to better estimate the size of the prognostic effect independent of tumour, node, metastasis staging. Given the substantial amount of heterogeneity identified, adherence to REMARK guidelines^25^ and adjustment of the survival analysis for known prognostic factors in future studies is recommended. Further, preregistration of such prognostic studies is important to help avoid the issues of reporting and publication bias.^64^ Using assumptions based on the findings of the present meta-analysis and a mutation frequency of 70%, we estimate a minimum of 433 patients with OAC (303 TP53 mutated and 130 TP53 wild-type) would be required to determine the effect of TP53 mutations on patient overall survival.

One approach to collect high-quality data is to include TP53 mutation analysis using targeted gene sequencing in the baseline analysis of trials of OAC therapies. If validated, TP53 analysis could be used to stratify patients in future trials. Stratifying patients based on TP53 mutation status may also have a role in clinical practice to guide treatment selection. For example, data suggest that TP53 mutation status may predict response to standard chemotherapeutic regimens such as fluorouracil or cisplatin,^65^ which has also recently been demonstrated in OAC.^66–68^ A prospective randomised trial in oesophageal cancer, aiming at determining this predictive effect of TP53 gene mutations (p53-Adjusted Neoadjuvant Chemotherapy for Potentially Resectable Esophageal Cancer; http://www.clinicalTrials.gov identifier NCT00525200; http://www.p53.at) has recently completed recruitment. Moreover, multiple therapeutic options directly targeting the TP53 gene are either currently in clinical trials or are already clinically available.^14^ ^18^ ^59^ For example, the first-in-class mutant p53 reactivator APR-246 has recently been shown to have significant antitumourigenic activity in OAC and synergises with DNA damaging agents such as cisplatin and 5-fluorouracil.^69^ As recent next-generation sequencing studies have demonstrated a high frequency of TP53 mutations in OAC of 70%,^19–21^ it seems likely that a TP53-directed therapeutic approach would be worthwhile for patients with this highly fatal cancer.

In summary, OAC remains one of the few GI malignancies for which molecular information is still not used to guide patient management. This study suggests that TP53 gene mutations have a clinically important negative prognostic impact on patients with OAC, which is relevant in light of recent genomic findings highlighting a central role of this gene in OAC pathogenesis and drugs currently in development and testing that directly target this gene. High-quality studies with large patient cohorts using modern sequencing technologies for TP53 mutation analysis are needed to confirm the independent prognostic effect of this frequent gene mutation.

## References

[1] Pohl H, Welch HG The role of overdiagnosis and reclassification in the marked increase of esophageal adenocarcinoma incidence. J Natl Cancer Inst 2005;97:142–6. 10.1093/jnci/dji024 15657344

[2] Clemons N, Phillips W, Lord RV Signaling pathways in the molecular pathogenesis of adenocarcinomas of the esophagus and gastresophageal junction. Cancer Biol Ther 2013;14 10.4161/cbt.25362 PMC3909547 23792587

[3] Arnold M, Soerjomataram I, Ferlay J, et al Global incidence of oesophageal cancer by histological subtype in 2012. Gut 2015;64:381–7. 10.1136/gutjnl-2014-308124 25320104

[4] Edgren G, Adami HO, Weiderpass E, et al A global assessment of the oesophageal adenocarcinoma epidemic. Gut 2013;62:1406–14. 10.1136/gutjnl-2012-302412 22917659

[5] Eheman C, Henley SJ, Ballard-Barbash R, et al Annual Report to the Nation on the status of cancer, 1975–2008, featuring cancers associated with excess weight and lack of sufficient physical activity. Cancer 2012;118:2338–66. 10.1002/cncr.27514 22460733 PMC4586174

[6] Brown LM, Devesa SS, Chow WH Incidence of adenocarcinoma of the esophagus among white Americans by sex, stage, and age. J Natl Cancer Inst 2008;100:1184–7. 10.1093/jnci/djn211 18695138 PMC2518165

[7] Thrift AP, Shaheen NJ, Gammon MD, et al Obesity and risk of esophageal adenocarcinoma and Barrett's esophagus: a mendelian randomization study. J Natl Cancer Inst 2014;106:pii: dju252 10.1093/jnci/dju252 PMC4200028 25269698

[8] Enzinger PC, Mayer RJ Esophageal cancer. N Engl J Med 2003;349:2241–52. 10.1056/NEJMra035010 14657432

[9] Rustgi AK, El-Serag HB Esophageal carcinoma. N Engl J Med 2014;371:2499–509. 10.1056/NEJMra1314530 25539106

[10] van Hagen P, Hulshof MC, van Lanschot JJ, et al Preoperative chemoradiotherapy for esophageal or junctional cancer. N Engl J Med 2012;366:2074–84. 10.1056/NEJMoa1112088 22646630

[11] Weaver JM, Ross-Innes CS, Fitzgerald RC The ‘-omics’ revolution and oesophageal adenocarcinoma. Nat Rev Gastroenterol Hepatol 2014;11:19–27. 10.1038/nrgastro.2013.150 23982683

[12] Lane DP Cancer. p53, guardian of the genome. Nature 1992;358:15–16. 10.1038/358015a0 1614522

[13] Petitjean A, Mathe E, Kato S, et al Impact of mutant p53 functional properties on TP53 mutation patterns and tumor phenotype: lessons from recent developments in the IARC TP53 database. Hum Mutat 2007;28:622–9. 10.1002/humu.20495 17311302

[14] Levine AJ, Oren M The first 30 years of p53: growing ever more complex. Nat Rev Cancer 2009;9:749–58. 10.1038/nrc2723 19776744 PMC2771725

[15] Aas T, Borresen AL, Geisler S, et al Specific P53 mutations are associated with de novo resistance to doxorubicin in breast cancer patients. Nat Med 1996;2:811–14. 10.1038/nm0796-811 8673929

[16] O'Shea D, O'Riain C, Taylor C, et al The presence of TP53 mutation at diagnosis of follicular lymphoma identifies a high-risk group of patients with shortened time to disease progression and poorer overall survival. Blood 2008;112:3126–9. 10.1182/blood-2008-05-154013 18628487 PMC2954748

[17] Young KH, Leroy K, Moller MB, et al Structural profiles of TP53 gene mutations predict clinical outcome in diffuse large B-cell lymphoma: an international collaborative study. Blood 2008;112:3088–98. 10.1182/blood-2008-01-129783 18559976 PMC2569165

[18] Olivier M, Hainaut P, Barresen-Dale AL. Prognostic and predictive value of TP53 mutations in human cancer. In: Hainaut P, Wiman K 25 years of p53 research. Dordrecht, The Netherlands: Springer, 2005:320–8.

[19] Dulak AM, Stojanov P, Peng S, et al Exome and whole-genome sequencing of esophageal adenocarcinoma identifies recurrent driver events and mutational complexity. Nat Genet 2013;45:478–86. 10.1038/ng.2591 23525077 PMC3678719

[20] Nones K, Waddell N, Wayte N, et al Genomic catastrophes frequently arise in esophageal adenocarcinoma and drive tumorigenesis. Nat Commun 2014;5:5224 10.1038/ncomms6224 25351503 PMC4596003

[21] Weaver JM, Ross-Innes CS, Shannon N, et al Ordering of mutations in preinvasive disease stages of esophageal carcinogenesis. Nat Genet 2014;46:837–43. 10.1038/ng.3013 24952744 PMC4116294

[22] Soussi T, Beroud C Assessing TP53 status in human tumours to evaluate clinical outcome. Nat Rev Cancer 2001;1:233–40. 10.1038/35106009 11902578

[23] Kaye PV, Haider SA, James PD, et al Novel staining pattern of p53 in Barrett's dysplasia--the absent pattern. Histopathology 2010;57:933–5. 10.1111/j.1365-2559.2010.03715.x 21166706

[24] Guyatt GH, Oxman AD, Vist GE, et al GRADE: an emerging consensus on rating quality of evidence and strength of recommendations. BMJ 2008;336:924–6. 10.1136/bmj.39489.470347.AD 18436948 PMC2335261

[25] McShane LM, Altman DG, Sauerbrei W, et al REporting recommendations for tumor MARKer prognostic studies (REMARK). Nat Clin Pract Oncol 2005;2:416–22. 10.1038/ncponc0252 16130938

[26] Hayden JA, van der Windt DA, Cartwright JL, et al Assessing bias in studies of prognostic factors. Ann Intern Med 2013;158:280–6. 10.7326/0003-4819-158-4-201302190-00009 23420236

[27] Higgins JPT GSe. Cochrane Handbook for Systematic Reviews of Interventions Version 5.1.0[updated March 2011], 2011.

[28] Parmar MK, Torri V, Stewart L Extracting summary statistics to perform meta-analyses of the published literature for survival endpoints. Stat Med 1998;17:2815–34. 10.1002/(SICI)1097-0258(19981230)17:24<2815::AID-SIM110>3.0.CO;2-8 9921604

[29] Tierney JF, Stewart LA, Ghersi D, et al Practical methods for incorporating summary time-to-event data into meta-analysis. Trials 2007;8:16 10.1186/1745-6215-8-16 17555582 PMC1920534

[30] DerSimonian R, Laird N Meta-analysis in clinical trials. Control Clin Trials 1986;7:177–88. 10.1016/0197-2456(86)90046-2 3802833

[31] Higgins JP, Thompson SG, Deeks JJ, et al Measuring inconsistency in meta-analyses. BMJ 2003;327:557–60. 10.1136/bmj.327.7414.557 12958120 PMC192859

[32] Thompson SG, Sharp SJ Explaining heterogeneity in meta-analysis: a comparison of methods. Stat Med 1999;18:2693–708. 10.1002/(SICI)1097-0258(19991030)18:20<2693::AID-SIM235>3.0.CO;2-V 10521860

[33] Altman DG, Bland JM Interaction revisited: the difference between two estimates. BMJ 2003;326:219 10.1136/bmj.326.7382.219 12543843 PMC1125071

[34] Egger M, Davey Smith G, Schneider M, et al Bias in meta-analysis detected by a simple, graphical test. BMJ 1997;315:629–34. 10.1136/bmj.315.7109.629 9310563 PMC2127453

[35] R: A language and environment for statistical computing[program]. Vienna, Austria: R Foundation for Statistical Computing, 2013.

[36] Wickham H ggplot2: elegant graphics for data analysis. New York: Springer, 2009.

[37] Viechtbauer W Conducting Meta-Analyses in R with the metafor Package. J Stat Softw 2010;36:1–48. 10.18637/jss.v036.i03

[38] Max Gordon TL forestplot: Advanced Forest Plot Using ‘grid’ Graphics. Secondary forestplot: Advanced Forest Plot Using ‘grid’ Graphics 2015.

[39] Flejou JF, Paraf F, Potet F, et al p53 protein expression in Barrett's adenocarcinoma: a frequent event with no prognostic significance. Histopathology 1994;24:487–9. 10.1111/j.1365-2559.1994.tb00561.x 8088724

[40] Duhaylongsod FG, Gottfried MR, Iglehart JD, et al The significance of c-erb B-2 and p53 immunoreactivity in patients with adenocarcinoma of the esophagus. Ann Surg 1995;221:677–83; discussion 83–4 10.1097/00000658-199506000-00007 7794072 PMC1234694

[41] Wu TT, Watanabe T, Heitmiller R, et al Genetic alterations in Barrett esophagus and adenocarcinomas of the esophagus and esophagogastric junction region. Am J Pathol 1998;153:287–94. 10.1016/S0002-9440(10)65570-8 9665490 PMC1852949

[42] Ireland AP, Shibata DK, Chandrasoma P, et al Clinical significance of p53 mutations in adenocarcinoma of the esophagus and cardia. Ann Surg 2000;231:179–87. 10.1097/00000658-200002000-00005 10674608 PMC1420984

[43] Schneider PM, Stoeltzing O, Roth JA, et al P53 mutational status improves estimation of prognosis in patients with curatively resected adenocarcinoma in Barrett's esophagus. Clin Cancer Res 2000;6:3153–8. 10955797

[44] Falkenback D, Nilbert M, Oberg S, et al Prognostic value of cell adhesion in esophageal adenocarcinomas. Dis Esophagus 2008;21:97–102. 10.1111/j.1442-2050.2007.00749.x 18269642

[45] Madani K, Zhao R, Lim HJ, et al Prognostic value of p53 mutations in oesophageal adenocarcinoma: final results of a 15-year prospective study. Eur J Cardio Thoracic Surg 2010;37:1427–32. 10.1016/j.ejcts.2009.12.018 20227286

[46] Lehrbach DM, Cecconello I, Ribeiro U Jr, et al Adenocarcinoma of the esophagogastric junction: relationship between clinicopathological data and p53, cyclin D1 and Bcl-2 immunoexpressions. Arq Gastroenterol 2009;46:315–20. 10.1590/S0004-28032009000400013 20232013

[47] Cavazzola LT, Rosa AR, Schirmer CC, et al Immunohistochemical evaluation for P53 and VEGF (Vascular Endothelial Growth Factor) is not prognostic for long term survival in end stage esophageal adenocarcinoma. Revista do Colegio Brasileiro de Cirurgioes 2009;36:24–34. 10.1590/S0100-69912009000100007 20076865

[48] Sauter ER, Keller SM, Erner SM p53 correlates with improved survival in patients with esophageal adenocarcinoma. J Surg Oncol 1995;58:269–73. 10.1002/jso.2930580414 7723372

[49] Soontrapornchai P, Elsaleh H, Joseph D, et al TP53 gene mutation status in pretreatment biopsies of oesophageal adenocarcinoma has no prognostic value. Eur J Cancer 1999;35:1683–7. 10.1016/S0959-8049(99)00172-0 10674013

[50] Ribeiro U Jr, Finkelstein SD, Safatle-Ribeiro AV, et al p53 sequence analysis predicts treatment response and outcome of patients with esophageal carcinoma. Cancer 1998;83:7–18. 10.1002/(SICI)1097-0142(19980701)83:1<7::AID-CNCR2>3.0.CO;2-R 9655287

[51] Aloia TA, Harpole DH Jr., Reed CE, et al Tumor marker expression is predictive of survival in patients with esophageal cancer. Ann Thorac Surg 2001;72:859–66. 10.1016/S0003-4975(01)02838-7 11565671

[52] Gibson MK, Abraham SC, Wu TT, et al Epidermal growth factor receptor, p53 mutation, and pathological response predict survival in patients with locally advanced esophageal cancer treated with preoperative chemoradiotherapy. Clin Cancer Res 2003;9:6461–8. 14695149

[53] Fareed KR, Al-Attar A, Soomro IN, et al Tumour regression and ERCC1 nuclear protein expression predict clinical outcome in patients with gastro-oesophageal cancer treated with neoadjuvant chemotherapy. Br J Cancer 2010;102:1600–7. 10.1038/sj.bjc.6605686 20461087 PMC2883154

[54] Kandioler D, Schoppmann SF, Zwrtek R, et al The biomarker TP53 divides patients with neoadjuvantly treated esophageal cancer into 2 subgroups with markedly different outcomes. A p53 Research Group study. J Thorac Cardiovasc Surg 2014;148:2280–6. 10.1016/j.jtcvs.2014.06.079 25135238

[55] Rice TW, Blackstone EH, Rusch VW 7th edition of the AJCC Cancer Staging Manual: esophagus and esophagogastric junction. Ann Surg Oncol 2010;17:1721–4. 20369299 10.1245/s10434-010-1024-1

[56] Findlay JM, Middleton MR, Tomlinson I A systematic review and meta-analysis of somatic and germline DNA sequence biomarkers of esophageal cancer survival, therapy response and stage. Ann Oncol 2015;26:624–44. 10.1093/annonc/mdu449 25214541 PMC4374384

[57] Chen M, Huang J, Zhu Z, et al Systematic review and meta-analysis of tumor biomarkers in predicting prognosis in esophageal cancer. BMC Cancer 2013;13:539 10.1186/1471-2407-13-539 24206575 PMC3828582

[58] Berg JS, Khoury MJ, Evans JP Deploying whole genome sequencing in clinical practice and public health: meeting the challenge one bin at a time. Genet Med 2011;13:499–504. 10.1097/GIM.0b013e318220aaba 21558861

[59] Olivier M, Taniere P Somatic mutations in cancer prognosis and prediction: lessons from TP53 and EGFR genes. Curr Opin Oncol 2011;23:88–92. 10.1097/CCO.0b013e3283412dfa 21045690

[60] Edlund K, Larsson O, Ameur A, et al Data-driven unbiased curation of the TP53 tumor suppressor gene mutation database and validation by ultradeep sequencing of human tumors. Proc Natl Acad Sci USA 2012;109:9551–6. 10.1073/pnas.1200019109 22628563 PMC3386058

[61] Akiyama J, Alexandre L, Baruah A, et al Strategy for prevention of cancers of the esophagus. Ann N Y Acad Sci 2014;1325:108–26. 10.1111/nyas.12529 25266020

[62] Belkadi A, Bolze A, Itan Y, Whole-genome sequencing is more powerful than whole-exome sequencing for detecting exome variants. Proc Natl Acad Sci USA 2015;112:5473–8. 10.1073/pnas.1418631112 25827230 PMC4418901

[63] Hayden JA, Cote P, Bombardier C Evaluation of the quality of prognosis studies in systematic reviews. Ann Intern Med 2006;144:427–37. 10.7326/0003-4819-144-6-200603210-00010 16549855

[64] Altman DG The time has come to register diagnostic and prognostic research. Clin Chem 2014;60:580–2. 10.1373/clinchem.2013.220335 24520099

[65] Weller M Predicting response to cancer chemotherapy: the role of p53. Cell Tissue Res 1998;292:435–45. 10.1007/s004410051072 9582400

[66] Heeren PA, Kloppenberg FW, Hollema H, et al Predictive effect of p53 and p21 alteration on chemotherapy response and survival in locally advanced adenocarcinoma of the esophagus. Anticancer Res 2004;24:2579–83. 15330218

[67] Beardsmore DM, Verbeke CS, Davies CL, et al Apoptotic and proliferative indexes in esophageal cancer: predictors of response to neoadjuvant therapy[corrected]. J Gastrointest Surg 2003;7:77–86; discussion 86–7 10.1016/S1091-255X(02)00141-5 12559188

[68] van Olphen SH, Biermann K, wijnhoven bP, et al Sa1926: SOX2 and p53 Protein Expression Predicts Response to Preoperative Chemoradiotherapy in Patients With Esophageal Adenocarcinoma. Gastroenterology 2015;148 (Suppl1):S-357.

[69] Liu DS, Read M, Cullinane C, et al APR-246 potently inhibits tumour growth and overcomes chemoresistance in preclinical models of oesophageal adenocarcinoma. Gut 2015;64:1506–16. 10.1136/gutjnl-2015-309770 26187504

