# Supplementary material for: The prognostic value of TP53 mutations in oesophageal adenocarcinoma: a systematic review and meta-analysis
Source: Gut. 2016 Jan 5;66(3):399–410. doi: 10.1136/gutjnl-2015-310888 (PMC5534764; doi:10.1136/gutjnl-2015-310888)
Supplement: Supplementary materials [file gutjnl-2015-310888supp001.pdf]

SUPPLEMENTARY DATA

SUPPLEMENTARY FILE 1

Summary Findings of IARC TP53 Database Analysis

The most current R17 version of the IARC TP53 database contains three studies[42, 51, 66] that report on the prognostic effect of TP53 mutations in esophageal adenocarcinoma cohorts, all of which are included in our meta-analysis.

In 345 EACs compiled in the IARC TP53 database, the most frequently occurring mutations were G:C to A:T transitions at CpG sites (43.5% of tumors) in exons 5, 7, 8 (Supplementary Figure 1A) with a mean mutation frequency in any single nucleotide at this site of 1.27 (SD 0.03). The most frequent effect of this type of mutation was missense mutations (found in 78% of p53 mutations; Supplementary Figure 1B).

Of all the tumors included in the database, 245 had information on their immunohistochemistry staining pattern. Missense mutations most frequently caused positive immuno-staining and occurred most commonly within the L2/L3, L1/S/H2 and NDBL/beta-sheet protein domains (Supplementary Figure 1C and 1D). However, approximately 27% of TP53 mutant tumors showed negative immuno-staining patterns (false negatives), as these are frequently deletion mutations (Supplementary Figure 2).

SUPPLEMENTARY FIGURE LEGENDS

Supplementary Figure 1. Analysis of EAC patients (n = 345) in the IARC TP53 mutation database. Supplementary Figure 1A depicts the frequency of types of TP53 gene mutations as well as their genetic location. Supplementary Figure 1B shows the resulting effects of these mutations on protein isotypes. Supplementary Figure 1C shows the corresponding IHC staining patterns of the type of TP53 gene mutations, where 1D shows how the TP53 mutation effect affects IHC staining patterns (EAC n = 245).

Supplementary Figure 2. Analysis of IARC TP53 database to determine frequency of interpretations of immunohistochemistry staining patterns in the presence of TP53 gene mutations.

Supplementary Figure 3. Funnel plot of all studies included in the present meta-analysis for assessment of possible publication bias.

Supplementary Figure 4. Forest plot of the effect of TP53 on survival stratified by histology and adjustment for standard prognostic variables, all studies.

Supplementary Figure 5. Forest plot of effect of TP53 on survival only including studies performing TP53 gene sequencing before (supplemenary figure 5A) and after sensitivity analysis (removal of Gibson et al.; 5B). Supplementary figure 5C depicts the forest plot of studies with pure EAC cohorts that performed TP53 gene sequencing.

Supplementary Figure 6. Forest plot of meta-regression analysis of study factors associated inter-study heterogeneity. Depicted are the effect esimates (solid squares) of change in log HR and corresponding 95% confidence interval (95%CI, solid line). Total (solid diamond) represents the overall effect estimate (log HR) of mutant TP53 and corresponding 95%CI as calculated from all 16 included studies.

Supplementary Figure 1.

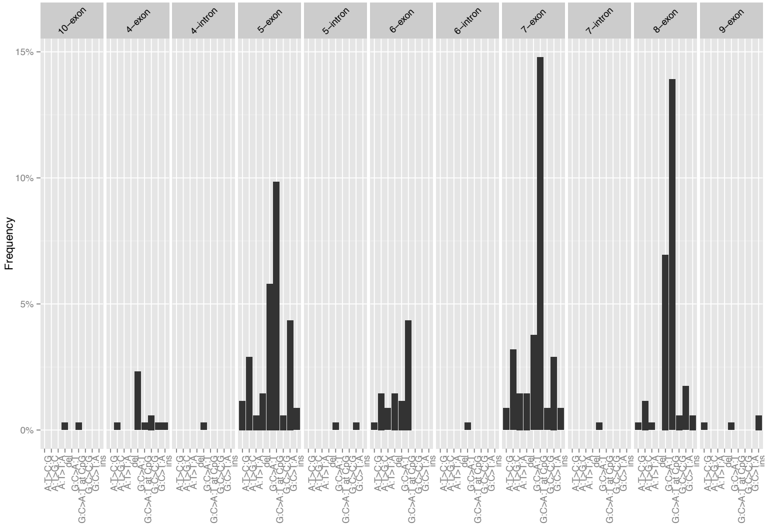

A TP53 Mutations & Gene Location

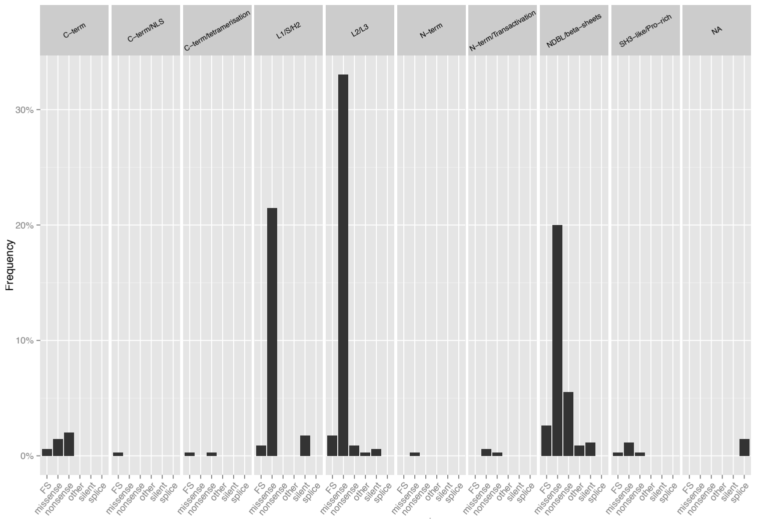

B TP53 Mutation Effects and Resulting p53 Protein Isotypes

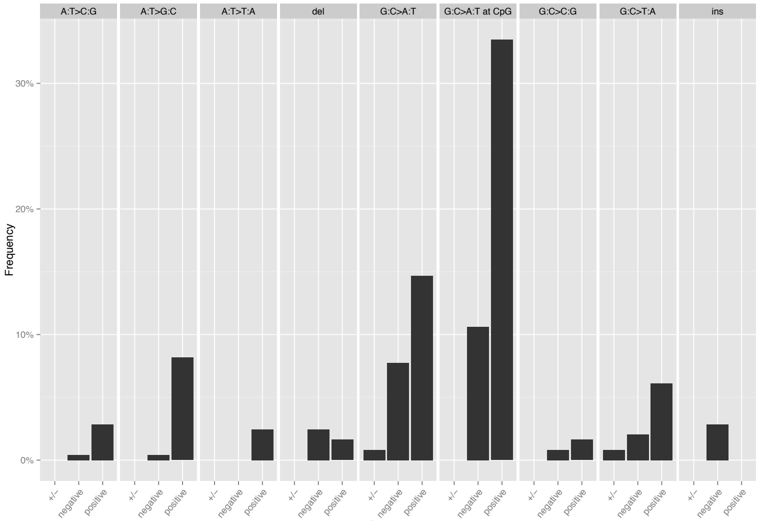

C TP53 Mutation Types & Immunohistochemistry

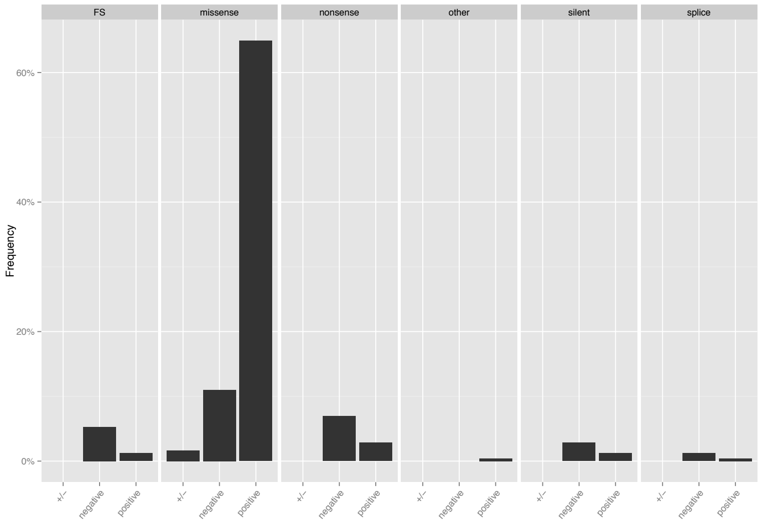

D TP53 Mutation Effects & Immunohistochemistry

Supplementary Figure 2.

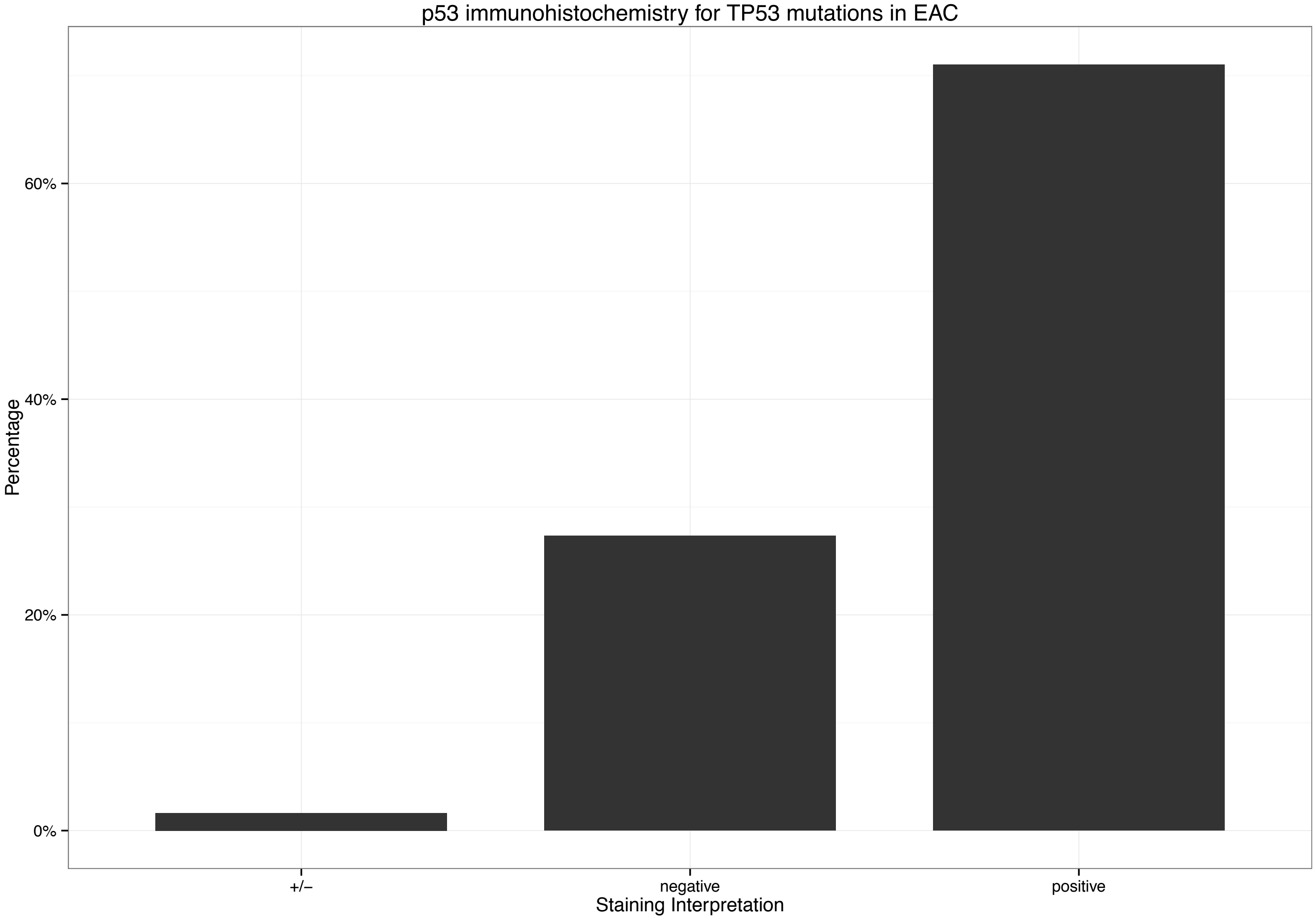

Supplementary Figure 3.

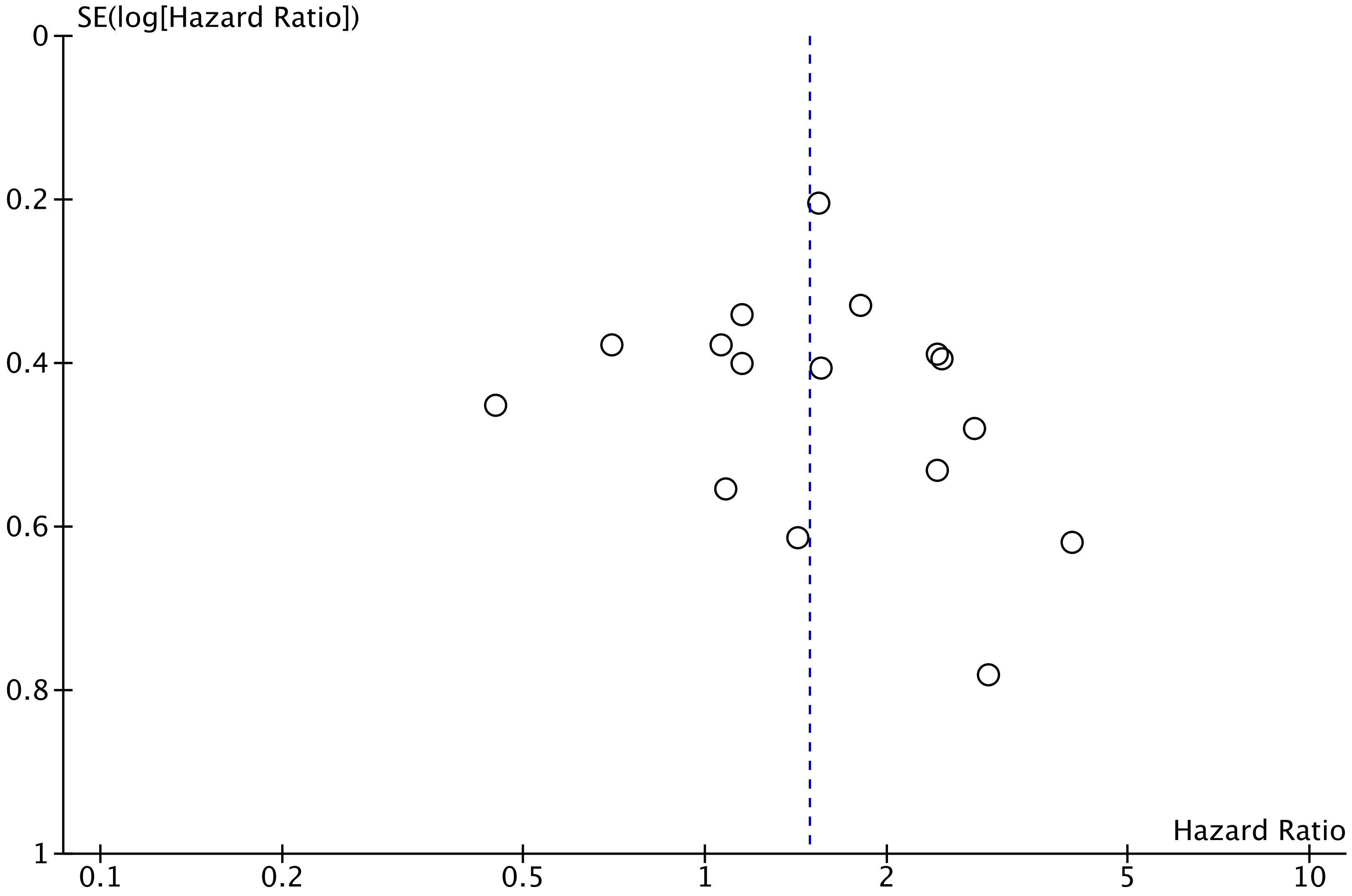

Supplementary Figure 4.

Study or Subgroup	log[Hazard Ratio]	SE	Weight	Hazard Ratio IV, Random, 95% CI
**3.27.1 Unadjusted HR estimates and mixed histology**				
Fareed	0.14	0.342	8.2%	1.15 [0.59, 2.25]
Gibson	−0.792	0.452	5.6%	0.45 [0.19, 1.10]
Ribeiro	1.024	0.479	5.2%	2.78 [1.09, 7.12]
**Subtotal (95% CI)**			**19.0%**	**1.12 [0.44, 2.85]**
Heterogeneity: $Tau^2 = 0.50$; $Chi^2 = 7.63$, df = 2 (P = 0.02); $I^2 = 74\%$				
Test for overall effect: Z = 0.24 (P = 0.81)				
**3.27.2 Unadjusted HR estimates and EAC only cohorts**				
Duhaylongsod	0.077	0.555	4.1%	1.08 [0.36, 3.21]
Fléjou	−0.351	0.378	7.2%	0.70 [0.34, 1.48]
Lehrbach	0.443	0.406	6.6%	1.56 [0.70, 3.45]
Madani	0.432	0.204	13.2%	1.54 [1.03, 2.30]
Sauter	1.08	0.781	2.3%	2.94 [0.64, 13.61]
Soontrapornchai	0.139	0.4	6.7%	1.15 [0.52, 2.52]
**Subtotal (95% CI)**			**40.2%**	**1.32 [1.00, 1.75]**
Heterogeneity: $Tau^2 = 0.00$; $Chi^2 = 4.81$, df = 5 (P = 0.44); $I^2 = 0\%$				
Test for overall effect: Z = 1.94 (P = 0.05)				
**3.27.3 Adjusted HR estimates and mixed histology**				
Aloia	0.88	0.389	6.9%	2.41 [1.12, 5.17]
Kandioler	1.4	0.62	3.5%	4.06 [1.20, 13.67]
**Subtotal (95% CI)**			**10.4%**	**2.79 [1.46, 5.33]**
Heterogeneity: $Tau^2 = 0.00$; $Chi^2 = 0.50$, df = 1 (P = 0.48); $I^2 = 0\%$				
Test for overall effect: Z = 3.12 (P = 0.002)				
**3.27.4 Adjusted HR estimates and EAC only cohorts**				
Cavazzola	0.357	0.612	3.5%	1.43 [0.43, 4.74]
Falkenback	0.06	0.377	7.2%	1.06 [0.51, 2.22]
Ireland	0.88	0.53	4.4%	2.41 [0.85, 6.81]
Schneider	0.904	0.396	6.8%	2.47 [1.14, 5.37]
Wu	0.588	0.331	8.5%	1.80 [0.94, 3.44]
**Subtotal (95% CI)**			**30.5%**	**1.72 [1.20, 2.48]**
Heterogeneity: $Tau^2 = 0.00$; $Chi^2 = 2.99$, df = 4 (P = 0.56); $I^2 = 0\%$				
Test for overall effect: Z = 2.92 (P = 0.004)				
**Total (95% CI)**			**100.0%**	**1.48 [1.16, 1.90]**
Heterogeneity: $Tau^2 = 0.08$; $Chi^2 = 22.43$, df = 15 (P = 0.10); $I^2 = 33\%$				
Test for overall effect: Z = 3.12 (P = 0.002)				
Test for subgroup differences: $Chi^2 = 5.18$, df = 3 (P = 0.16), $I^2 = 42.1\%$				

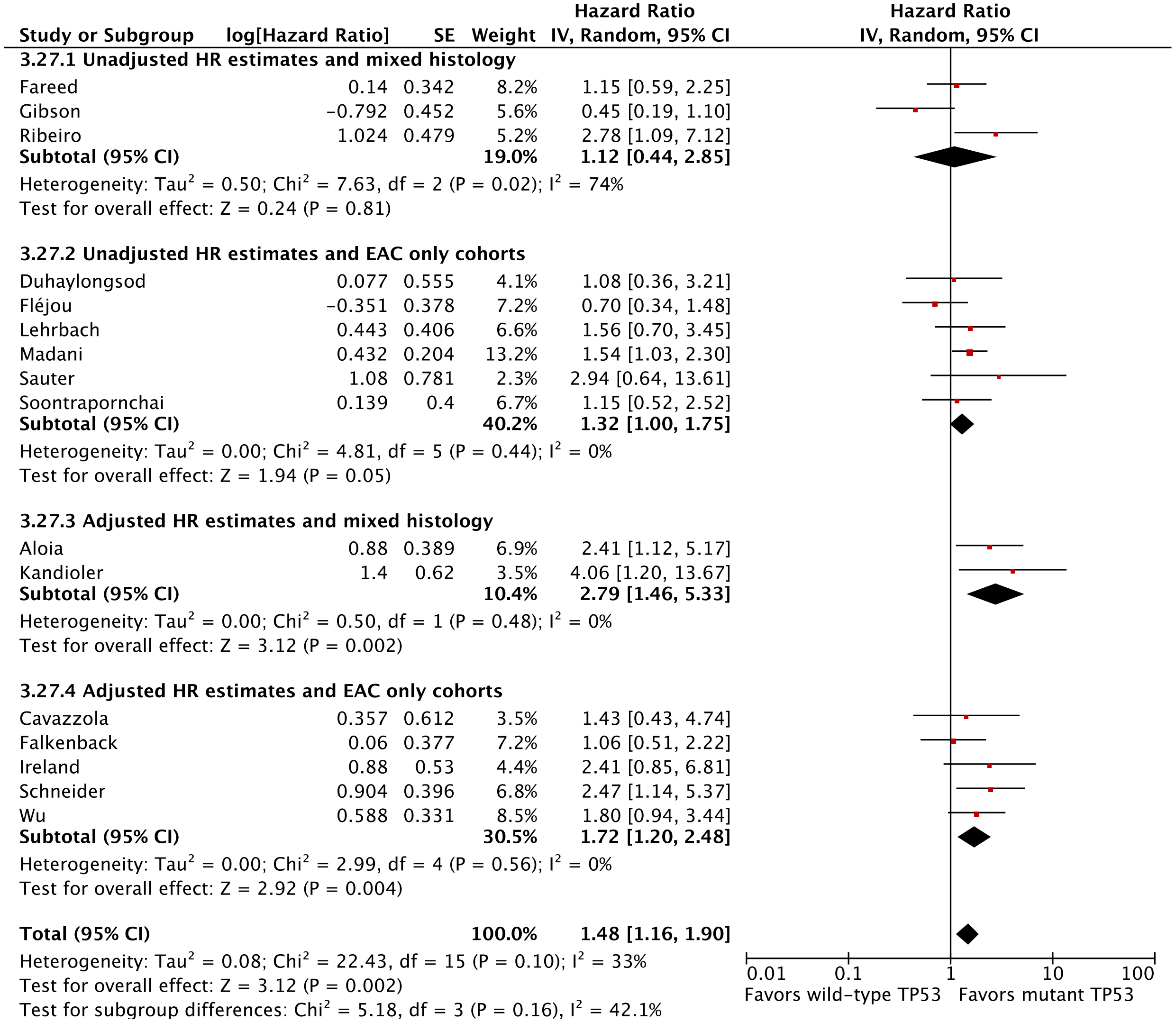

Supplementary Figure 5.

A

Study or Subgroup	log[Hazard Ratio]	SE	Weight	Hazard Ratio IV, Random, 95% CI	Year
Ribeiro	1.024	0.479	15.4%	2.78 [1.09, 7.12]	1998
Schneider	0.904	0.396	18.0%	2.47 [1.14, 5.37]	2000
Ireland	0.88	0.53	13.9%	2.41 [0.85, 6.81]	2000
Gibson	−0.792	0.452	16.2%	0.45 [0.19, 1.10]	2003
Madani	0.432	0.204	24.8%	1.54 [1.03, 2.30]	2009
Kandioler	1.4	0.62	11.7%	4.06 [1.20, 13.67]	2014
**Total (95% CI)**			**100.0%**	**1.80 [1.05, 3.08]**	

Heterogeneity: $Tau^2 = 0.26$; $Chi^2 = 13.13$, df = 5 (P = 0.02); $I^2 = 62\%$
Test for overall effect: Z = 2.13 (P = 0.03)

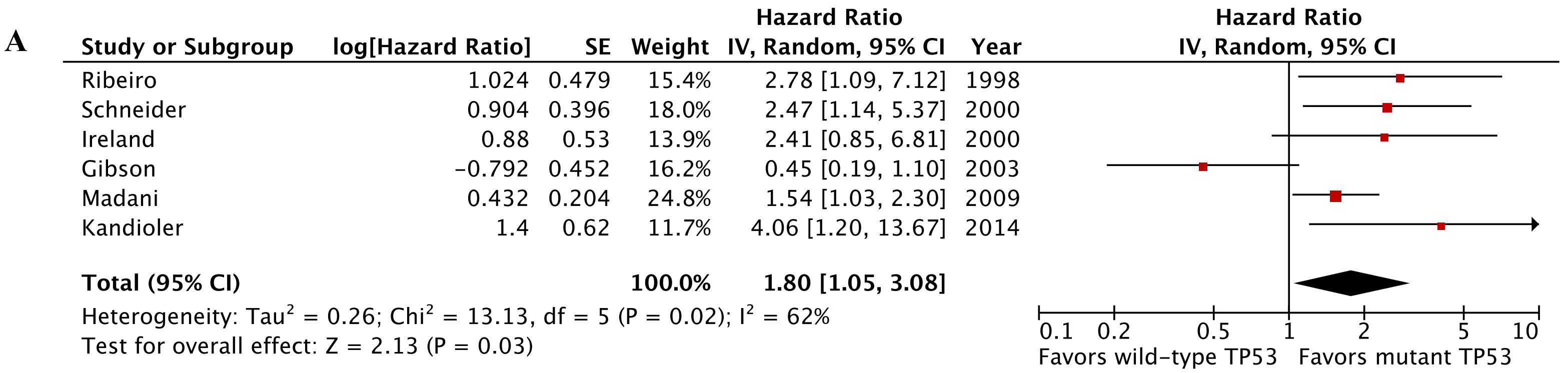

B

Study or Subgroup	log[Hazard Ratio]	SE	Weight	Hazard Ratio IV, Random, 95% CI	Year
Ribeiro	1.024	0.479	10.6%	2.78 [1.09, 7.12]	1998
Ireland	0.88	0.53	8.7%	2.41 [0.85, 6.81]	2000
Schneider	0.904	0.396	15.6%	2.47 [1.14, 5.37]	2000
Madani	0.432	0.204	58.7%	1.54 [1.03, 2.30]	2009
Kandioler	1.4	0.62	6.4%	4.06 [1.20, 13.67]	2014
**Total (95% CI)**			**100.0%**	**1.95 [1.44, 2.65]**	

Heterogeneity: $Tau^2 = 0.00$; $Chi^2 = 3.80$, df = 4 (P = 0.43); $I^2 = 0\%$
Test for overall effect: Z = 4.28 (P < 0.0001)

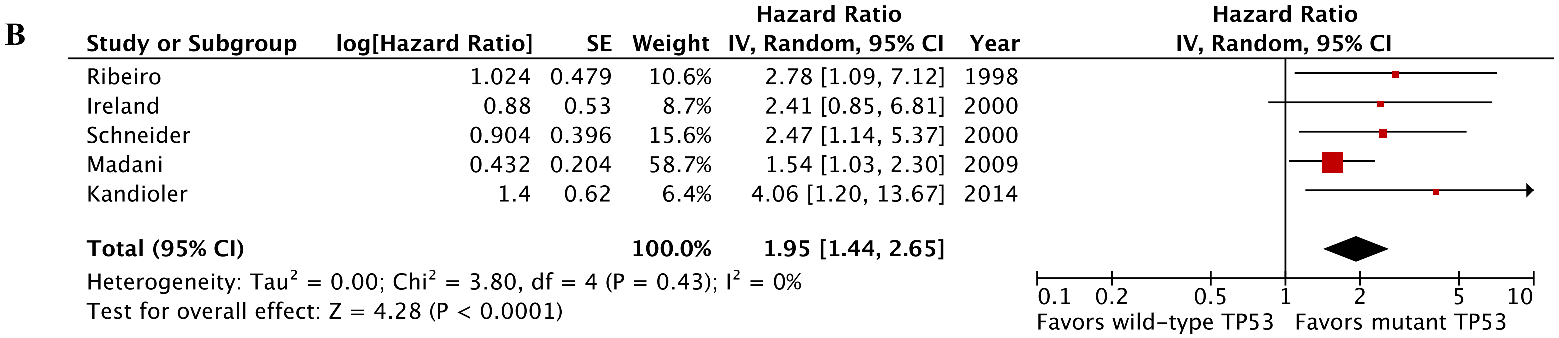

C

Study or Subgroup	log[Hazard Ratio]	SE	Weight	Hazard Ratio IV, Random, 95% CI	Year
Ireland	0.88	0.53	10.5%	2.41 [0.85, 6.81]	2000
Schneider	0.904	0.396	18.8%	2.47 [1.14, 5.37]	2000
Madani	0.432	0.204	70.7%	1.54 [1.03, 2.30]	2009
**Total (95% CI)**			**100.0%**	**1.76 [1.26, 2.47]**	

Heterogeneity: $Tau^2 = 0.00$; $Chi^2 = 1.51$, df = 2 (P = 0.47); $I^2 = 0\%$
Test for overall effect: Z = 3.31 (P = 0.0009)

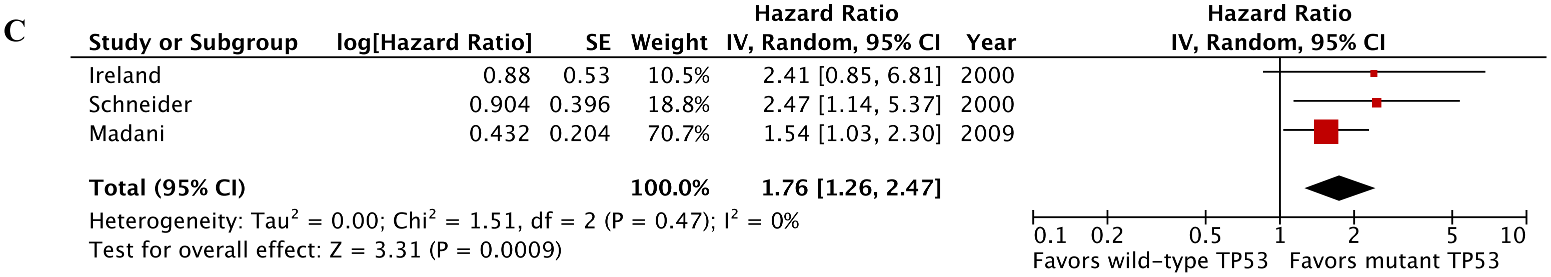

Supplementary Figure 6.

Study variable	Number of studies	logHR change estimate	95% CI
Pure EAC cohort	16	-0.0670	-0.629–0.496
No immunohistochemistry	16	0.2440	-0.248–0.735
Adjusted HR estimates	16	0.4590	0.011–0.908
Risk of bias = low	16	0.5800	0.058–1.102
Number of patients	16	-0.0020	-0.009–0.007
Study cohort age (mean/median)	14	-0.0960	-0.183–-0.009
Surgical specimens analysed	15	-0.0420	-0.643–0.56
Only surgically treated patients	16	-0.2010	-1.044–0.643
CRTx included	15	-0.0590	-0.541–0.424
Neo-adjuvant CRTx included	15	-0.1900	-0.649–0.27
Percentage p53 mutated	16	-0.0180	-0.039–0.003
Stage 4 included in analysis	11	-0.4670	-1.217–0.284
p53 mutated EAC more LN	12	0.1970	-0.438–0.831
p53 mutated EAC higher stage	12	0.1140	-0.494–0.723
**Total**	**16**	**0.3941**	**0.151–0.638**

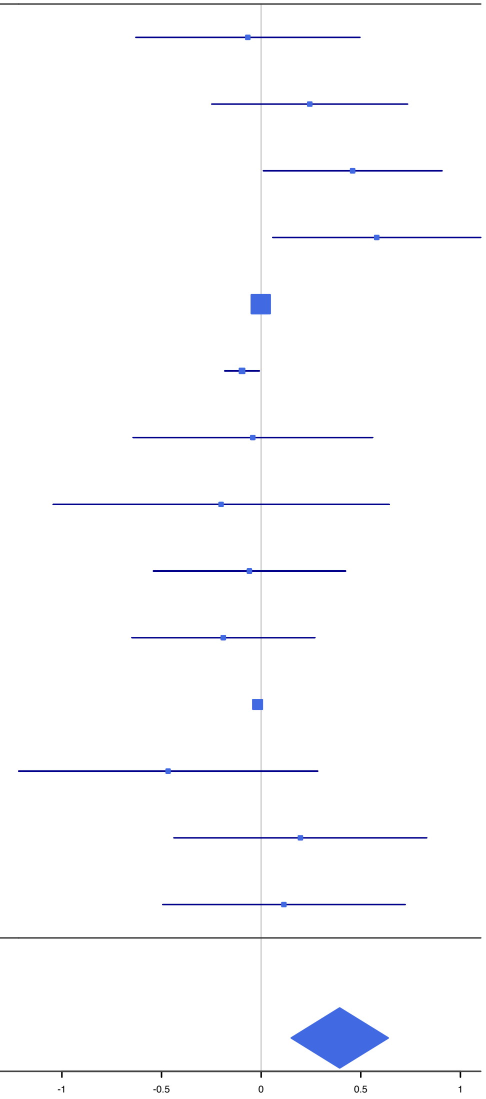

Supplementary Table 1. Clinicopathological and survival data of all studies included in final meta-analysis.

Reference	N	Tx/Tis	T1	T1a	T1b	T2	T3	T4	N0	N+	N1	N2	Mx	M1	G1	G2	G3	Stage 0	Stage I	Stage II	Stage III	Stage IV	TNM Stage Edition	R0	R1	R2	RX	Median Survival Total (months)	Median Survival TP53 "mutated"	Median Survival TP53 "wild-type"
Fléjou[38]	62	-	20	8	12	7	35	-	30	32	32	-	-	-	25	20	17	-	19	14	23	6	-	-	-	-	-	28.0	15	15
Duhaylongsod[39]	42	-	-	-	-	-	-	-	28	14	-	-	-	-	-	-	-	-	-	-	-	-	-	-	-	-	-	-	19.8	18.2
Sauter[47]	24	-	-	-	-	-	-	-	8	8	-	-	-	-	15	-	8	-	-	-	-	-	-	-	-	-	-	-	28.0	13.0
Wu[40]	92	-	-	-	-	-	-	-	-	-	-	-	-	-	-	-	-	-	11	24	42	15	4	-	-	-	-	16.3	10	23
Ribeiro[49]	42	7	8	-	-	8	12	-	19	18	18	-	-	1	4	15	13	7	4	17	8	1	4	-	-	-	-	-	21.6	40.2
Soontrapornchai[48]	135	-	-	-	-	-	-	-	20	34	-	-	-	21	5	34	33	-	-	-	-	-	5	-	-	-	-	-	12.0	14.0
Schneider[42]	59	-	-	-	-	-	-	-	-	-	-	-	-	-	4	19	36	0	22	18	12	3	4	49	4	2	4	-	-	-
Ireland[41]	37	-	5	3	2	7	19	-	7	24	10	14	-	2	4	13	14	-	-	-	-	-	-	-	-	-	-	-	10	28
Aloia[50]	61	-	31	-	-	14	16	-	61	-	-	-	-	-	-	-	-	-	31	30	0	0	5	61	0	0	0	38.3	18.0	49.0
Gibson[51]	54	-	-	-	-	-	-	-	-	-	-	-	-	-	6	33	15	-	-	-	-	-	-	-	-	-	-	-	-	20.7
Falkenback[43]	54	4	16	-	-	7	32	-	36	23	23	-	-	1	-	-	-	4	16	17	21	1	6	-	-	-	-	41.7	41.7	39.8
Madani[44]	142	-	35 (T1 + T2)	-	-	-	107 (T2 + T3)	-	55	87	87	-	-	0	41	37	64	0	12	49	76	5	-	119	17	6	-	20.0	16	25
Cavazzola[46]	46	-	6	-	-	6	13	13	18	20	20	-	1	3	6	12	20	-	5	9	20	4	-	38	0	0	-	70.4	58.1	63.2
Lehrbach[45]	75	-	4	-	-	27	44	0	21	54	54	-	-	5	-	-	-	-	14	15	30	16	6	-	-	-	-	-	21.5	29.5
Fareed[52]	245	2	18	-	-	72	139	14	62	183	-	-	-	2	-	-	-	-	-	-	-	-	-	-	-	-	-	-	26.7	53.2
Kandioler[53]	36	3	5	-	-	7	13	8	10	17	17	-	-	3	5	13	8	-	-	-	-	-	-	24	3	-	-	13.9	8.6	26.2

Supplementary Table 2. Subgroup analyses					
**Subgroup**	**Number of studies**	**Number of patients**	**Pooled HR (95% CI)**	***p*-value**	**Heterogeneity I^2 statistic (*p*-value)**
**Assay and histology type**					
Immunohistochemistry only	8	417	1.28 (0.95 – 1.73)	0.10	0% (0.43)
Immunohistochemistry only and pure EAC cohorts	6	290	1.14 (0.79 – 1.66)	0.49	0% (0.57)
No immunohistochemistry	8	471	1.68 (1.14 – 2.47)	0.009	50% (0.05)
No immunohistochemistry after sensitivity analysis	7	425	1.82 (1.40 – 2.36)	<0.0001	0% (0.50)
No immunohistochemistry and pure EAC cohorts	5	354	1.68 (1.27 – 2.22)	0.0003	0% (0.64)
Sequencing only studies	6	330	1.80 (1.05 – 3.08)	0.03	62% (0.02)
Sequencing only studies after sensitivity analysis	5	284	1.95 (1.44 – 2.65)	<0.0001	0% (0.43)
Sequencing only studies and pure EAC cohorts	3	213	1.76 (1.26 – 2.47)	0.0009	0% (0.47)
**Adjustment for tumor stage**					
Studies including HRs adjusting for standard prognostic variables	7	327	1.94 (1.41 – 2.66)	<0.0001	0% (0.53)
Studies including HRs adjusting for standard prognostic variables *and* pure EAC only cohorts	5	258	1.72 (1.20 – 2.48)	0.004	0% (0.56)
Studies with unadjusted HRs for standard prognostic variables	9	533	1.22 (0.88 – 1.70)	0.24	38% (0.12)
Studies with unadjusted HRs for standard prognostic variables, *but* pure EAC cohorts	6	386	1.32 (1.00 – 1.75)	0.05	0% (0.44)
**Risk of bias and tumor type**					
Low risk of bias	4	197	2.29 (1.50 – 3.48)	0.0001	0% (0.70)
Low risk of bias and EAC only cohorts	3	161	2.11 (1.35 – 3.31)	0.001	0% (0.80)
High and unclear risk of bias	12	691	1.29 (0.98 – 1.70)	0.07	30% (0.15)

High risk of bias *but* pure EAC cohort	8	488	1.29 (1.00 – 1.67)	0.05	0% (0.64)
Table 3 continued.					
**Subgroup**	**Number of studies**	**Number of patients**	**Pooled HR (95% CI)**	***p*-value**	**Heterogeneity I^2 statistic (*p*-value)**
**Other exploratory subgroups of interest**					
Only chemo-radiotherapy naïve patients (regardless of risk of bias)	6	397	1.60 (1.21 – 2.11)	0.0009	0% (0.71)
Only chemo-radiotherapy naïve patients (regardless of risk of bias) *and* pure EAC only cohorts	5	336	1.50 (1.11 – 2.02)	0.0008	0% (0.80)
Only studies with no neo-adjuvant chemo-radiotherapy (regardless of risk of bias)	7	446	1.68 (1.29 – 2.18)	0.0001	0% (0.67)
Only studies with no neo-adjuvant chemo-radiotherapy *and* pure EAC cohorts (regardless of risk of bias)	6	385	1.60 (1.21 – 2.11)	0.0009	0% (0.69)
